# Solitary Living Brings a Decreased Weight and an Increased Agility to the Domestic Silkworm, *Bombyx mori*

**DOI:** 10.3390/insects12090809

**Published:** 2021-09-09

**Authors:** Zhenglin Zhu, Yuting Tan, Siyu Xiao, Zhufen Guan, Wen Zhao, Zhijun Dai, Gexin Liu, Ze Zhang

**Affiliations:** Laboratory of Evolutionary and Functional Genomics, School of Life Sciences, Chongqing University, Chongqing 401331, China; tanyuting202108@163.com (Y.T.); 202026021017@cqu.edu.cn (S.X.); 17835424450@139.com (Z.G.); zhaowenyx@163.com (W.Z.); 18716433548@163.com (Z.D.); 15123887928@163.com (G.L.)

**Keywords:** solitary, loneliness, group living, weight, agility

## Abstract

**Simple Summary:**

We identified and validated that solitary living brings a decreased weight and an increased agility to silkworms. Solitary silkworms have a faster movement in response to food or physical stress than group-living counterparts. These contradict previous thoughts that solitary or lonely life is always harmful to animals or humans. We identified differently expressed genes (DEGs) and microRNAs (DEmiRNAs) resulted from solitary living. These DEGs and DEmiRNAs are functionally associated with the phenotypic changes led by solitary living.

**Abstract:**

The domestic silkworms, *Bombyx mori*, always live in groups and little is known of the outcomes of solitary living. We bred solitary silkworms and performed a comprehensive investigation of the difference between solitary and group-living silkworms. The results show that solitary silkworms had significantly lower weights than group-living counterparts. Moreover, solitary silkworms had faster movements under food luring or heat stress than the group-living ones, supported by extensive behavior experiments. These findings inferred that an increased agility resulted from solitary living. For an understanding of the molecular mechanism associated with solitary living, we performed integrated mRNA and miRNA (microRNA) sequencing of tissues for solitary and group-living silkworms. We identified 165 differently expressed genes (DEGs) and 6 differently expressed miRNAs between the solitary and group-living silkworms. Functional and pathway analyses indicated that these DEGs are associated with weight loss and agility increase. These findings compose a sketch depicting an association between the phenotypes and genes resulted from solitary living and refresh the understanding of solitary living and loneliness, which has an increased prevalence in our modern society.

## 1. Introduction

With the growing population aging and demographic changes, loneliness or social isolation has become increasingly prevalent in our modern society [1]. Loneliness is a social problem, as it damages physical health, leading to an increase in depression behaviors, and is a risk factor for morbidity and mortality [2,3,4,5]. The investigation of loneliness and associated molecular mechanisms is important. The clinical research is time-consuming and has several limitations, such as medical ethics. Aside from humans, animals also show depression behaviors when leading a solitary or lonely life [1,6,7]. Comparative studies of solitary and group-living animals and the associated molecular machinery [8,9] are instructive for understanding the effects of loneliness on human health. For animals, solitude is not always disadvantageous and has benefits, such as a reduced social transmission of parasites, a decreased susceptibility to infection and an avoidance of social competition [10,11,12,13,14]. Solitary bees reduce investment in communication compared to their social relatives [15]. The isolated lifestyle led by humans during national lockdowns efficiently controlled the transmission of COVID-19 [16]. Solitary/group living is an important environmental factor. The interactions between this factor and behaviors deserve investigation [17].

In breeding animals, group living has benefits, including increased feeding efficiency and lower mass loss [18]. The investigation of solitary animals has potential industrial applications. Some animals, such as the migratory locust, are capable of transiting from a solitary phase to a group/gregarious state [19], where the chemosensory protein genes and the takeout gene LmigTO1 modulate the switch [20]. It is possible to use these switch-modulating genes to prevent the transition of the migratory locust from a solitary to gregarious state (devastating to agriculture plants).

**Figure 1:**
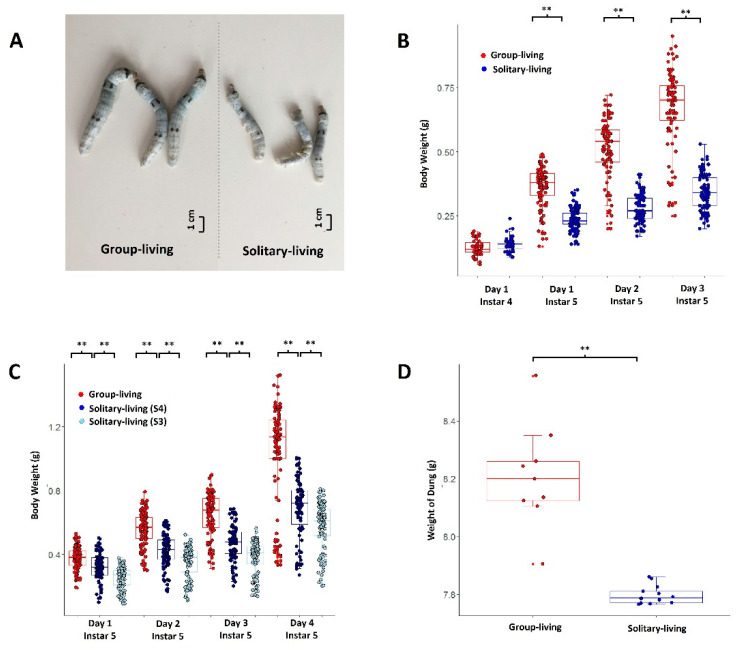
Evidence showing that solitary-living silkworms experienced a loss of body weight compared to group-breeding silkworms. (**A**) is a photo showing the difference in appearance of solitary and group-living silkworms in the 3rd day of the 5th instar. (**B**) shows the body weights of solitary (blue) and group-living (red) silkworms from the 1st day to the 3rd of the 5th instar. The weights on the 1st day of the 4th instar acts as a control for comparison. (**C**) is the comparison of the body weight of group-living (red), S4 (solitarily bred as early as the 1st day of the 4th instar, dark blue) and S3 (solitarily bred as early as the 1st day of the 3rd instar, light blue) solitary-living silkworms. Weights are recorded from the 1st day to the 4th day of the 5th instar. (**D**) is the comparison of the weight of dungs between solitary (blue) and group-living (red) silkworms. The weight of the dungs of six silkworms on the 3rd day of the 5th instar are weighed and recorded each time. In B to D, ‘**’ denotes *p*-value < 0.01. Statistics are performed by Wilcox test.

**Figure 2:**
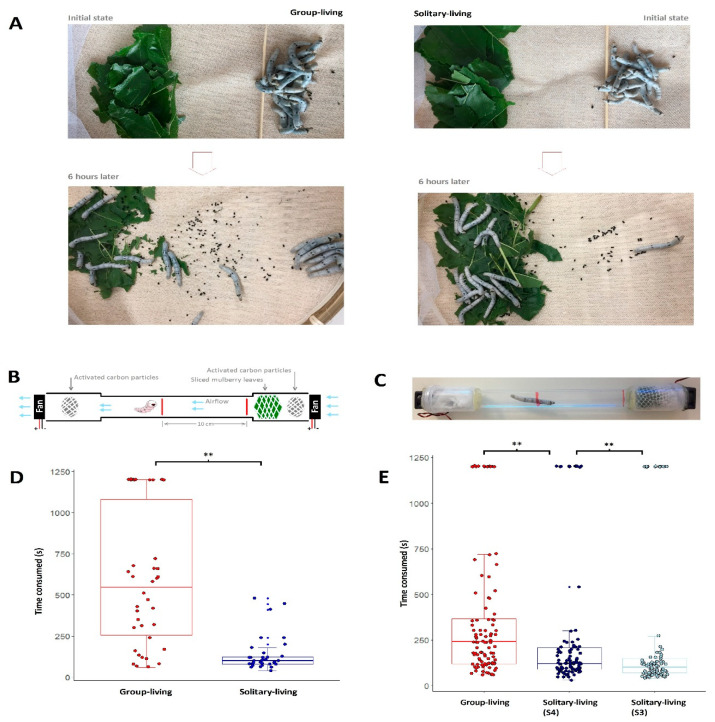
The difference in time taken to move under food luring between solitary and group-living silkworms. (**A**) is an experiment with a group of solitary/group-living silkworms and a packet of mulberry leaves on a bamboo basket. After six hours, 15/16 solitary and 9/17 group-living silkworms arrived at mulberry leaves. B is a sketch showing the details of the device used to test the movement of a silkworm under food luring. (**C**) is a picture of a real product of (**B**). (**D**) shows the difference in time taken to move between solitary and group-living silkworms. E shows the difference in time taken to move for group-living silkworms, S4 and S3 solitary silkworms. The meanings of S3 and S4 follow Figure 1. In (**D**,**E**), ‘**’ denotes *p*-value < 0.01. Statistics are performed by Wilcox test.

**Figure 3:**
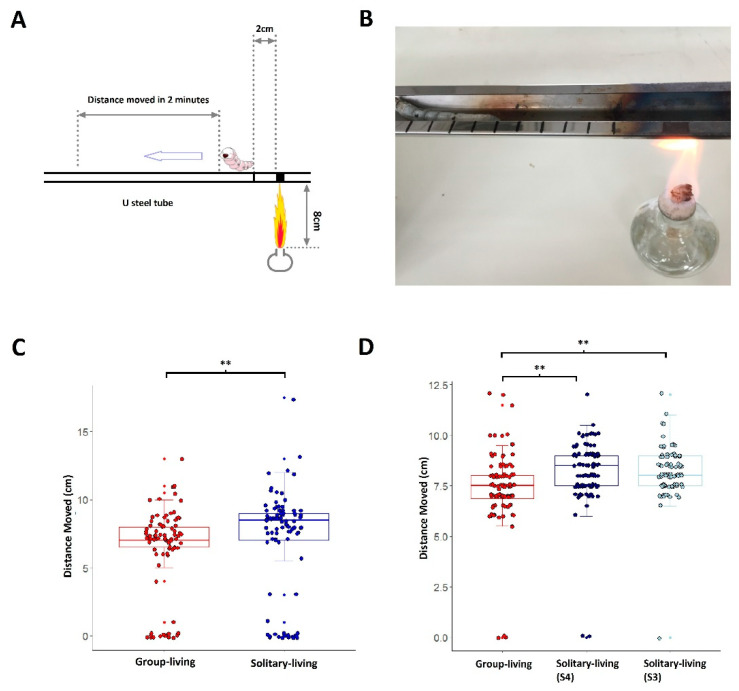
The difference in distance moved under heat stress between solitary and group-living silkworms. A is a sketch showing the details of the device used to test the movement of a silkworm under heat stress. (**B**) is a picture of a real product of (**A**). (**C**) shows the difference in the distance moved between solitary and group-living silkworms. (**D**) shows the distance moved for group-living silkworms, S3 and S4 solitary silkworms. In (**C**,**D**), ‘**’ denotes *p*-value < 0.01. Statistics are performed by Wilcox test.

**Figure 4:**
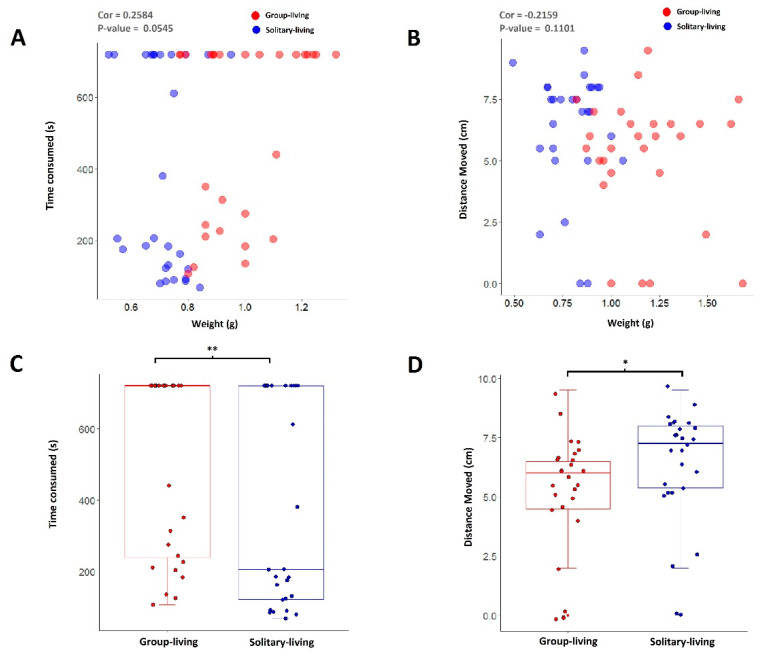
Correlation analysis between the weight of solitary/group-living silkworms and their performance in food luring or heat stress experiments. (**A**) is a scatterplot showing the correlation of the time taken to move and the body weight of solitary (blue)/group-living silkworms (red). (**B**) is a scatterplot showing the distance moved to evade heat stress and the body weight of solitary (blue) and group-living silkworms (red). (**C**) is a comparison of the time taken to move (corresponding to (**A**)) between solitary and group-living silkworms. (**D**) is a comparison of the distance moved (corresponding to (**B**)) to evade heat stress between solitary and group-living silkworms. The results of correlation analyses are shown at the head of each scatterplot. In (**C**,**D**), ‘*’ denotes *p*-value < 0.05 and ‘**’ denotes *p*-value < 0.01. Statistics are performed by Wilcox test.

The silkworm, *Bombyx mori*, is domesticated from its wild ancestry, *Bombyx mandarina*. *B. mandarina* leads a solitary life in the wild, but *B. mori* is always bred in groups in the silk industry. Compared to group living, solitary living has benefits for *B. mandarina*, such as the avoidance of competition and prey from natural enemies. The group-breeding of *B. mori* creates convenience for feeding. Whether solitary-breeding silkworms, *B. mori*, show a different phenotype compared to group-breeding silkworms is unknown. For this issue, we intentionally bred solitary and group-living silkworms, then evaluated the potential phenotypic difference and associated molecular mechanisms through behavioral experiments, transcriptome analyses and miRNA (microRNA) analyses. We identified interesting changes in phenotypes (a loss of body weight and an increase in agility) from group living to solitary living (Figure 1, Figure 2, Figure 3, Figure 4 and Figures S1–S5) and associated molecular mechanisms (potentially relevant genes, miRNAs and pathways), providing a further clarification of the biological effects of solitary living or loneliness.

## 2. Materials and Methods

### 2.1. Solitary Breeding

For solitary breeding, we kept a single silkworm in a container (solitary-breeding container) nearly a hundred square centimeters large. For group breading, we kept tens of silkworms in a big container (group-breeding container). We threw in an excess of fresh mulberry leaves into the solitary-breeding container four times a day to ensure that the food for solitary-living silkworms was fresh and plentiful. For equality in the comparison, we also fed group-breeding silkworms four times a day and provided an excess of fresh mulberry leaves. We cleaned the solitary- and group-breeding containers every day. To prevent the mulberry leaves from drying and withering, we covered the solitary- and group-breeding containers with plastic sheets.

The silkworm larvae at instars 1 and 2 are tiny and would die easily under solitary breeding conditions. Thus, solitary-breeding silkworms were raised from the first day of the third instar or from the first day of the fourth instar, respectively. At the first day of the third instar or the first day of the fourth instar, we randomly selected silkworms and put them into solitary-breeding containers one by one. For the S3 solitary-breeding silkworms, nearly 12 days of solitary-living life passed until they were tested at the third day of the fifth instar. For the S4 solitary-breeding silkworms, nearly 8 days of solitary living passed until they were tested on the third day of the fifth instar.

### 2.2. Behavior Experiments

The test of silkworms’ response to food was performed in an indoor environment without airflow passing through. A rough test was performed using a bamboo basket covered by gauze. Solitary and group-living silkworms were randomly selected and placed into two bowls. Then, the solitary and group-living silkworms were poured onto designated positions in the bamboo basket. Bamboo picks were used to keep silkworms stationary in one position at the beginning. Silkworms were kept without food supply for one day before the behavior experiments. This was important in order to promote the silkworms’ willingness to move for food. Silkworms are always reluctant to move and like to stand still. Bamboo picks were extracted when the timing began. In principle, silkworms should have been lured by the food odor and should have started to move towards mulberry leaves. We recorded the positions of silkworms once per hour. For equality, the two packets of mulberry leaves used to lure solitary and group-living silkworms had the same weight and volume.

We also designed and made a tube-like device to test the responses of solitary and group-living silkworms. The device was an acrylic tube connected by a pair of plastic bottles at both ends. Newly produced devices were put in an airy place for nearly a month in order for them to be unscented. A pair of fans were equipped at two ends of the device. The fans, driven by 5 V DC, were controlled by a small PCB board, the power of which was provided by 220 V AC. Fans created airflow within the device. To prevent disturbance by outer chaotic odors, activated carbon particles wrapped with gauze squares were placed near the fans in the device. In the experiment, sliced mulberry leaves were packed by cotton mesh and used to emit odors to attract the silkworms located 10 cm away. The starting and the terminal positions were marked for convenience in recording. In the test, a packet of fresh mulberry leaf slices were first tucked into the device and then the fans were electrified. The cap at the terminal far from the mulberry leaf slices was opened, and a solitary or group-living silkworm was placed into the device. The silkworm was placed at the position near the starting position by a bamboo pick and then the cap was swiftly covered and tightened when the timing began. If a silkworm attracted by the odor from mulberry leaves arrived at the terminal near the mulberry packet within a limited time, e.g. 20 min, the time taken was recorded accordingly. If a silkworm did not touch the terminal position 20 min later, the experiment was terminated and the time consumed was recorded as 20 min. This was to avoid an endless wait in the situation that some silkworms stand still or move in a reverse direction. After the recording, the caps were opened, and the mulberry leaf slices were replaced to retain the freshness of the odor. When another silkworm was placed into the device, another round of test was started. Silkworms were not provided with food for one day before the experiment.

Moreover, we constructed another device to test silkworms’ responses to an instant environmental stress, e.g., intense heat. In this experiment, solitary or group-living silkworms were placed into a U-shape steel tube. At the beginning, the tube was heated by a spirit lamp at the position of 2 cm from the tail of the silkworm located at the starting position. Two minutes later, the distance moved was recorded. We cooled the tube for 5 min in ice after each test. We marked a scale in centimeters on the tube for convenience in recording.

We created a revised T-maze apparatus specialized for silkworm experiments. Following previous efforts [21], the device had two channels leading to a reward and punishment, respectively. There were two rooms in each channel. One room held liquid that emitted a specific odor (different in each channel) and the other room held mulberry leaves and a copper wire netting. The fans created airflows that brought the odor of a specific chemical and the odor of mulberry leaves towards the entrance of the apparatus, where a silkworm was placed in at the beginning of the test. Lured by the odor of mulberry leaves, the silkworm passed the entrance and made a selection of which channel to move into. There were two openings leading to two different channels, differentiated by different odors. One channel had a copper wire net with DC 250 V. A silkworm was punished if it moved into this channel. The copper wire net in the other channel did not have electricity, and a silkworm having moved into this channel was rewarded by mulberry leaves. We created an AC to DC converter to transform 220 V AC to 250 V DC, charging a capacitance of 250 V 100 μF, which supplied power for the wire net. There were holes capped at the top of the rooms which held chemical liquids. Chemical liquids could be added into the room through the holes after opening the cap. The workflow of the T-maze test was: (1) the electricity supply was switched on, (2) the mulberry leaf slices were added, (3) chemical odors were added, (4) a silkworm was placed inside the maze, (5) the electricity supply was switched off, (6) results were observed and recorded, (7) the silkworm was taken out, and (8) the mulberry leaf slices were taken out. Silkworms were kept without food supply for one day before the experiment.

### 2.3. mRNA and miRNA Sequencing

We extracted the tissues, including the brain, midgut and skin, of solitary and group-living silkworms at the third day of the fifth instar. The dissection of the silkworm took place on a cold silica gel plate. In the process of RNA extraction, lysed samples or homogenate were moved to room temperature for 5 to 10 min in preparation for the dissection of nuclear protein and nuclear acids. The homogenate was added by way of 0.2 mL of chloroform, vibrated heavily, put at room temperature for 3 min, and then centrifuged with 12,000× *g* rpm 4 °C for 10 min. The upper liquid was transferred into a clean centrifugal tube and isopropanol of the same volume as the transferred liquid was added, blended and kept at room temperature for 20 min. We performed centrifugation with 12,000× *g* rpm 4 °C for 10 min and discarded the supernatant. One milliliter of 75% ethanol was added to washout sediments. Then, we performed centrifugation with 12,000× *g* rpm 4 °C for 3 min, discarded the supernatant and put it in a dry place at room temperature for 5 to 10 min. An amount of 30–50 μL of RNase-free ddH_2_O was added to fully dissolve the RNA. The RNA solution was stored at −70 °C or used in mRNA or miRNA sequencing. We used Qubit2.0 to test the concentration of RNA and agarose gel electrophoresis to test the completeness of RNA and possible genome contamination. Sequencing was performed in the Illumina Platform (HiSeq X Ten). The 3′ adapter sequence was “TGGAATTCTCGGGTGCCAAGGAACTC”.

### 2.4. Identification of Differently Expressed Genes and miRNAs

We used the R package “ballgown” [22,23] to perform expression (FKPM) and sequencing quality analysis for the RNA-seq data in the three tissues, including the brain, midgut and cuticle, of solitary and group-living silkworms. We did not find a run with significant deviation or low quality in the 12 runs of data sequencing (Figures S6 and S7). Then, we carried out mapping of RNA-seq onto the silkworm reference genome [24] by Tophat [25] and performed transcriptome assembly and differential expression analysis by Cufflinks [26]. The pipeline includes the process of normalization and DEG (differently expressed genes) identification based on the FKPM values of transcripts. We required an adjusted FDR *p*-value < 0.05. In total, we obtained 27, 41 and 97 DEGs in the brain, midgut and cuticle, respectively.

For miRNA-seq analysis, we used cutadapt (cutadapt.readthedocs.io) to remove adapter sequences, wrote Python scripts and used R to perform statistics of read lengths (Figures S11–S13). We mapped reads onto the reference genome using bowtie2 [27] and used HTSeq [28] to count reads. We used the R package DEGseq [29] to find differently expressed miRNA (DEmiRNA). We carried out normalization of the expression data and searched for differently expressed sequences requiring |log2FoldChange| > 1 and adjusted *p*-value < 0.05. In total, we identified 12 differently expressed miRNA in the brain between solitary and group-living silkworms. In the same way, we performed differently expressed miRNA identification in the silkworm body with the brain excluded between solitary and group-living silkworms and finally obtained 9 cases. We performed miRNA target gene prediction by using four different software, including miRanda (www.microrna.org, accessed on 1st April 2019), Pita (genie.weizmann.ac.il, accessed on 1st April 2019), RNAhybrid [30] and TargetSpy [31]. We took the genes predicted by all four software as candidates. In this way, we identified 5924 candidate miRNA–gene pairs.

### 2.5. Functional Analysis

We used the R package clusterProfiler [32] to perform Gene Ontology (GO) enrichment analysis. The Entrez ID and GO of genes were downloaded from SGID [33], based on which we created a gene list for enrichment analysis. Gene information, including function prediction, subcellular location and signal peptide, were retrieved from SGID [33], in which the subcellular locations of proteins were predicted by CELLO v2.5 [34,35] and the signal peptides were predicted by SignalP [36,37].

### 2.6. Statistics

We performed Wilcoxon Rank Sum and Signed Rank tests (Wilcox test) to evaluate the statistical significance in comparing the behavior experimental data between solitary and group-living silkworms. The test for the association between paired samples was performed by R using the Pearson’s product moment correlation coefficient. We used Pearson’s chi-squared test (Chisq test) to evaluate the statistical significance of observed frequencies. In the Tophat pipeline, the normalization to identify DEG used the default option, classic-fpkm (fragments per kilobase of transcript per million mapped fragments) for Cufflinks and the geometric normalization method (default) for Cuffdiff. The FDR *p*-values and adjusted FDR *p*-values were as estimated by Cuffdiff’s statistical model of RNA-seq [25]. For the identification of DEmiRNA, DEGseq incorporated two methods to carry out normalization and DEmiRNA identification: an MA-plot-based method with a random sampling model and an MA-plot-based method with technical replicates [29]. The *p*-values were adjusted by multiple testing corrections [29]. In GO enrichment analysis, clusterProfiler normalized the dot sizes by count/(sum of each row) in the final plot.

## 3. Results

### 3.1. Solitary Living Brought a Loss of Body Weight

In the daily practice of breeding silkworms, individuals with a specific phenotype are selected and bred in an isolated environment. We observed that solitary individuals are always smaller in body size compared to group-living individuals. It is possibly solitary living that causes the loss of body weight. For a validation, we tried to evaluate the phenotypic difference between the silkworms (strain Dazao) intentionally bred in a solitary environment and those bred in groups. We kept hundreds of silkworms from the same parents in a group-living environment and randomly selected 100 individuals in the first day of the fourth instar. Of the 100 individuals, 50 were bred in a solitary environment and 50 were bred in a group-living environment. Plentiful food was provided in both breeding modes. The solitary silkworms were significantly lighter in the days from the first day to the third day of the fifth instar compared to group-living counterparts (Figure 1A,B). On the third day of the fifth instar, the weight of group-living silkworms (0.70 g median) was more than 50% higher than that of solitary silkworms (0.34 g median). Repeated experiments confirmed these results (Figure S1).

We also bred solitary silkworms (a total of 100) as early as the first day of the third instar (S3) and compared their weights with the silkworms bred in a solitary environment (a total of 100) as early as the first day of the fourth instar (S4) and the group-living silkworms (G; a total of 100). We found S3 samples were significantly lighter than S4, and S4 were significantly lighter than G (Figure 1C). These indicated that the longer silkworms have a solitary life, the lighter they will be. We did not start to raise solitary silkworms as early as the first instar or the second instar, for the silkworms at these stages are tiny and die easily in a solitary environment.

We weighed the dung excreted by solitary and group-living silkworms on the third day of the fifth instar. For convenience in comparison, we weighed the dung excreted by six silkworms at one time. Solitary silkworms excreted less dung than group-living counterparts (Figure 1D), inferring a decreased food input and an acquired anorexia for solitary silkworms. Solitary and group-living silkworms also showed differences in molting stages (Table S1).

### 3.2. Solitary-Living Silkworms Have an Increased Agility

We performed behavior experiments to compare solitary and group-living silkworms. First, we tried to test the movement of silkworms being lured by food, mulberry leaves. At the beginning, we put the solitary or group-living silkworms in one side and slices of mulberry leaves in another side, 10 cm away. Theoretically, silkworms should move towards the mulberry leaves. Six hours later, 15 out of 16 solitary silkworms and 9 out 17 group-living silkworms arrived at the place with food (Figure 2A). The difference is significant (Chisq test, *p*-value = 0.0085), suggesting an increased agility associated with solitary living. For a further validation, we performed a more rigorous experiment. We made a device to test the behaviors of silkworms under food luring (Figure 2B,C). The device was a transparent tube with wrapped activated carbon particles located at the ends for to avoid disturbance to the atmosphere in the device caused by outside odors. Fans equipped at the openings of the tube brought a unidirectional airflow across the mulberry leaves and the tested silkworm. The silkworm and the mulberry leaves were 10 cm apart at the beginning. Lured by the odor of mulberry leaves, the silkworm, in most cases, moved towards the mulberry leaves and finally stopped at the location with the mulberry leaves. We recorded the time taken to pass the 10 cm distance. In a few cases, silkworms stopped at the middle of the tube for more than tens of minutes. In order to avoid an unlimited waiting time, we set 20 min as the upper limit for recording. If a silkworm did not arrive at the terminal after 20 min, we terminated the test and recorded 20 min as the time taken. Solitary silkworms (100 s in median) took less time (Chisq test, *p*-value < 0.01, Figure 2D) than their group-living counterparts (545 s in median). We repeated the experiments and confirmed the results (Figure S2). We compared the performances of S3, S4 solitary and group-living silkworms. We found that the longer the solitary life is, the faster the silkworms move under food luring (Figure 2E).

Aside from food luring experiments, we made devices to test the behaviors of silkworms under physical stress. We put the silkworm into a steel tube (with a U-type cross section) and burned the steel at the position of 2 cm behind the silkworm (Figure 3A,B). The silkworm theoretically should move forward to escape being hurt by the heat. We recorded the distance moved in 2 min after the steel tube was burned. Solitary silkworms moved a significantly longer distance than group-living counterparts (*p*-value < 0.05, Figure 3C), also confirmed by repeated experiments (Figure S3). We performed the same experiments for S3 and S4 solitary silkworms but observed no significant difference. S3 and S4 both moved a longer distance than group-living silkworms (*p*-value < 0.01, Figure 3D).

Behavior experiments showed that solitary silkworms are more agile than group-living counterparts. Solitary silkworms are also lighter than group-living ones. To understand whether there is a relationship between the loss of body weight and the increase in agility, we recorded the body weight (W), the time taken to move under food luring (T) and the distance moved under heat stress (D) for each solitary or group-living silkworm. We set 12 min as the upper limit for recording in the food luring experiment, for a further reduction of the time spent to wait for motionless silkworms in the experiment. We did not find consistent significant correlations between W and T or between W and D (Figure 4 and Figure S4), suggesting a mutual independence between the loss of body weight and the increase in agility. 

For a further understanding of phenotypic changes after solitary living, we made a T-maize apparatus [21,38] to evaluate the difference in memory strength for solitary and group-living silkworms. In this experiment, the apparatus provided two choices combined with two different odors to the silkworm. One choice brought punishment, electronic shock, while the other choice brought rewards, mulberry leaves (Figure S5). Theoretically, the silkworm’s memory will construct a link between a selected odor and punishment/rewards the first time it experienced them. The silkworm with a good memory should move to the reward section and evade punishment the second time. The silkworm with bad memory will have a higher chance of receiving a punishment than those with good memory. We selected 4-methylcyclohexanol and the acetic acid as the liquid to produce odor, for the odors of these two chemicals have nearly identical attraction to silkworms (Table S2). These two odors were mixed with the odor of mulberry leaves to speed up the movement of silkworms. We performed the experiment four times, but did not find bias between solitary and group-living silkworms, not only in single cases (Tables S3 and S4), but also in an overall comparison of the ratios of the decreased counts of punishment to the counts of punishment (the item ‘Loss/Punishment’ in Table S4, Wilcox test, *p*-value = 0.5512).

### 3.3. Potential Molecular Mechanisms Associated with the Phenotypic Change Led by Solitary Living

**Figure 5:**
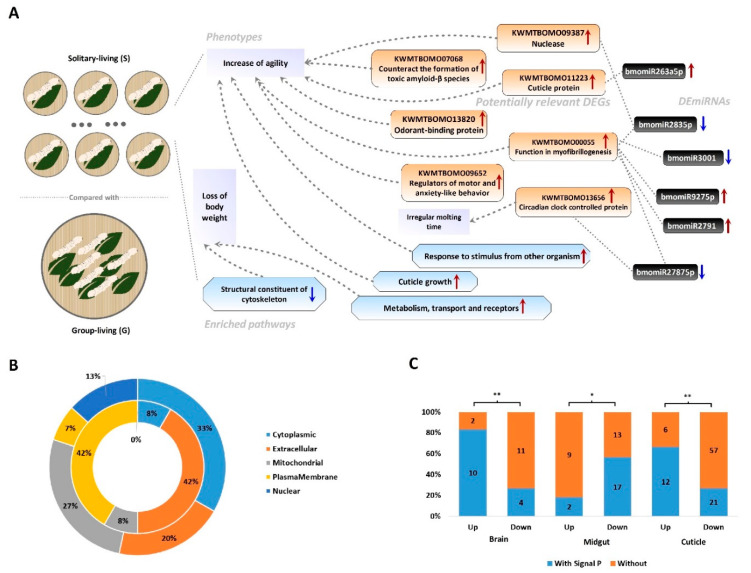
Transcriptome and miRNA analysis results. (**A**) is a predicted association network of the phenotypes induced by solitary living, potentially relevant DEGs, DEmiRNAs and enriched pathways. Red up-arrows denote up-regulation in solitary-living silkworms compared to group-living counterparts, while blue down-arrows denote down-regulation. DEGs, DEmiRNAs and enriched pathways are in boxes with different shapes and background colors. Dotted lines with arrows denote predicted contributions of DEGs or enriched pathways to phenotypes. Predicted associations between DEmiRNAs and DEGs are also indicated by dotted lines. The network is constructed based on Figure S8–S10 and Table S5–S9. (**B**) is the distribution of cell locations of DEGs up-regulated (inner cycle) and down-regulated (outer cycle) in the brain of solitary silkworms. (**C**) is the number/ratio of DEGs with signal peptides (Signal P) in brain, midgut and cuticle. Statistics are performed by Chisq test. ‘*’ denotes *p*-value < 0.05 and ‘**’ denotes *p*-value < 0.01.

To investigate the background molecular pathways and related genes that drive the change of body weight and agility in the transition from group-living to solitary living, we performed transcriptome sequencing of the brain, the midgut and the cuticle of solitary and group-living silkworms. The sequencing of the cuticle was for control. We also performed miRNA sequencing of the brain and the body (with the brain excised) aiming to evaluate the differences in regulation mechanisms in the brain between solitary and group-living silkworms. We identified 27 (Figure S8, Table S5), 41 (Table S6) and 96 (Table S7) DEGs in brain, midgut and cuticle. Twelve out of twenty-seven DEGs are up-regulated in the brain of solitary silkworms (solitary brain, for expression convenience). This percentage is higher than that in the midgut (11/41; Chisq test, *p*-value = 0.133) or in the cuticle (18/96; Chisq test, *p*-value = 0.006). We identified 12 DEmiRNA in the brain (Figure S9, Table S8) and 9 DEmiRNAs in the body (Table S9). The 12 and the 9 DEmiRNAs do not overlap, inferring the 12 DEmiRNAs are brain specific. We predicted the target gene of the 12 DEmiRNAs using four reported methods (for detail, see Materials and Methods) and obtained nine predicted DEmiRNA-DEG interaction pairs in brain (Table S5). Three DEGs up-regulated in the solitary brain are identified in correlation with three DEmiRNAs down-regulated in the solitary brain, respectively. This is in accordance with the fact that most miRNAs function in the negative regulation of genes [39,40]. The DEG, KWMTBOMO11223, predicted as a cuticle protein [33], is up-regulated in the solitary brain but associated with the DEmiRNA bmomiR263a5p, also up-regulated in the solitary brain. Potentially, this is a positive regulation [40]. A down-regulated brain DEG, KWMTBOMO00055, predicted to be a key component in the assembly and functioning of muscles [33], has five correlated DEmiRNAs (Table S5). The DEmiRNA bmomiR2835p are in relationship with three DEGs in the brain and is an important DEmiRNA, causing neurological alternations after solitary living (Figure 5A).

We obtained the annotation information of the functional and subcellular location of the DEGs from SGID [33] and performed statistics. Compared to down-regulated DEGs in the solitary brain, up-regulated DEGs showed a bias towards locating the plasma membrane and the extracellular region (Figure 5B), which are more important for neurocytes compared to other organelles. Moreover, most up-regulated DEGs in the solitary brain have signal peptides but down-regulated DEGs do not (Figure 5C), inferring a positive contribution of these up-regulated DEGs to neuronal development and physiology. Up-regulated DEGs in the midgut of solitary silkworms (solitary midgut for expression convenience) have fewer signal peptides than down-regulated DEGs. Up-regulated DEGs in the cuticle of solitary silkworms (solitary cuticle for expression convenience) also have more signal peptides than down-regulated DEGs, possibly resulting from the promoted physical activities in the neurocytes of solitary silkworms and associated with the increased sensitivity of solitary-living silkworms (Figure 5C).

We performed gene ontology (GO) enrichment analysis of all identified the DEGs. In accordance with behavior experiments, DEGs up-regulated in the solitary brain enriched in the function to respond to stimulus from other organisms (Figure S10A) possibly explained the increased agility of solitary silkworms. In the midgut, up-regulated DEGs in solitary silkworms enrich the metabolism, transport and receptor activities, while down-regulated DEGs enrich the cell structure (Figure S10B). These should be the causation of the loss of body weight under solitary living. DEGs in the solitary cuticle have down-regulation in immunity and up-regulation in the growth of cuticle (Figure S10C). In accordance with this, solitary-living silkworms in the lab are die more easily than group-living counterparts, possibly caused by the reduction in immune activity.

KWMTBOMO13820 is an up-regulated DEG in the solitary brain and predicted to be capable of binding to odorant (Table S5). The up-regulation of this gene may make the silkworm easier to smell the odor of food. KWMTBOMO09652 is a DEG predicted to express peptidoglycan recognition proteins (PGRPs). PGRPs are key sensing molecules of the innate immune system [41]. They are also regulators of motor and anxiety-like behavior in mice [42]. Previous work validated that PGRP knockout mice had changes in anxiety-behavior and reduced rearing [42]. Thus, PGRPs are potential key DEGs leading to phenotypic changes after solitary living. KWMTBOMO09387 is predicted to be a nuclease that cleaves cGAMP, which plays a key role in neurophysiology [43]. KWMTBOMO13656 is a DEGs in the brain predicted to function in circadian clock. In accordance, the time to molt for solitary silkworms are irregular, but that for group-living ones are regular (Table S1). KWMTBOMO07068 is predicted to be a lysozyme capable of counteracting the formation of toxic amyloid-β species for Alzheimer disease [44]. There are up-regulated DEGs with neurological functions in the solitary midgut (Table S6). These DEGs may impact the intake of food for solitary silkworms. As a summary of the above descriptions, we built a potential association network of DEGs, DEmiRNAs, enriched pathways and phenotypes induced by solitary living (Figure 5A).

## 4. Discussion

Through the performance of strict behavior experiments on the domestic silkworm, we validated their increased agility after solitary living. Solitary silkworms have a faster movement in response to food or physical stress than group-living silkworms. These contradicted previous thoughts that solitary or lonely life is always harmful to animals or humans [1,5,18]. However, we did not observe significant effects of solitary living on memory strength. Although solitary living benefits neuronal response, it brings weight loss and is disadvantageous for the immune system. On a molecular level, the solitary silkworms have a decline in the expression of immune-related genes (Table S6). In the practice of breeding silkworms, solitary-living silkworms are die easily and need more care than group-living ones. The weakening of the immune system also happens in other solitary animals [18,45,46]. Loneliness or an isolated life is also harmful to physical health in humans [1].

The loss of body weight after solitary living harms silk production but benefits survival in a wild environment. A silkworm with a big body size is more likely to be attacked than a silkworm with a small one. *B. mandarina*, the wild outgroup of the domestic silkworm, have a solitary life in all larva stages. Domestication is a process that makes the silkworms adapt to group-breeding for mass production. The breeding of domestic silkworms in a solitary-living environment is a reversal of the progress of domestication. In the process, it is plausible that some wildness is strengthened, such as an increased agility and a decreased body size. *B. mandarina* is smaller and more agile than the domestic silkworm, *B. mori*. However, it is nearly impossible to perform similar behavior experiments for *B. mandarina*, which is choosy in food (only taking in live leaves in a mulberry tree but refusing picked mulberry leaves) and always stays still when encountering danger or facing enemies.

There is a sharp loss of body weight after solitary living (Figure 1). Lone silkworms took less food than group-breeding ones. The decrease in the daily intake of food is possibly caused by neurological and molecular changes in the midgut. The former may lead to anorexia and the latter may cause a promotion of catabolism and a reduction in anabolism (Figure S10B). In the human world, loneliness relates to anorexia nervosa [47]. When keeping animals, group-breeding reduces the waste of food, and thus promotes feeding efficiency. It also increases the body weight of animals.

From the predicted association network of the phenotypes, DEGs, DEmiRNAs and enriched pathways (Figure 5A), we inferred that it is the elevation of the nervous, olfactory and motor systems, possibly led by the up-regulation of six or more relevant genes, that causes the increase in agility after solitary living. The alteration in gene expression may partly be driven by six or more miRNAs. Possibly, DNA methylation, DNA–protein interaction or N6-methyladenosine may also play a role in gene regulation, contributing to phenotypic changes after solitary living. Solitary-living silkworms have an increase in catabolism and a reduction in anabolism (Figure S10B), which possibly is associated with the elevation of the motor system. The up-regulation of anxiety-relevant genes, e.g., KWMTBOMO09652, resulted from solitary living should cause anorexia, in accordance with the observation that solitary-living silkworms have less excretion than group-living ones (Figure 1D). Thus, the elevation of catabolism and the up-regulation of anorexia-relevant genes should contribute to the weight loss of solitary-living silkworms. Solitary silkworms performed better in regard to movement in behavior experiments than group-living ones and reflected an increased agility, possibly also contributed by an up-regulation of genes relevant to the development of cuticle and muscle (Figure 5A and Figure S10) and a loss of body weight. These refreshed the knowledge of solitary and loneliness and should be instructive for future research in breeding animals and the medical treatment of brain diseases.

## 5. Conclusions

Our results identified and validated an increased agility and a loss of body weight associated with solitary living for the domestic silkworm, *Bombyx mori*. After the change from group living to solitary living, DEGs were identified with functions in neurophysiology and digestion. There were also DEmiRNAs predicted to be in relationship with those DEGs. Thus, a transfer from group living to solitary living altered the regulation of miRNAs and the expression of genes, ultimately resulting in a more agile and smaller silkworm than before, which is confirmed by functional and pathway analyses of these DEGs and DEmiRNA. The identified DEGs and DEmiRNAs are important both for further research of solitary/loneliness in medicine and the evolutionary history of domestic animals.

## Data Availability

The raw sequencing data have been deposited in the NCBI SRA database with the accession number: PRJNA748819.

## Supplementary Materials

The following are available online at https://www.mdpi.com/article/10.3390/insects12090809/s1, Figure S1: Repeated experiments to compare the body weight between solitary and group-living silkworms from the first day to the third day of the fifth instar, Figure S2: Repeated experiments to compare the time consumed to move under food luring between solitary and group-living silkworms, Figure S3: Repeated experiments to compare the distance moved under heat stress between solitary and group-living silkworms, Figure S4: Repeated experiments of the correlation analysis between the weight of solitary/group-living silkworms and the performance under food luring/heat stress, Figure S5: The apparatus used to test the memory strengths of silkworms, Figure S6: Statistics of RNA-seq data, Figure S7: The distribution of genes’ expression of log2 (FKPM + 1) in tissues, including brain, midgut and brain, Figure S8: The expression (FKPM) of DEGs in the brain between solitary (S) and group-living (G) silkworms, Figure S9: The heatmap of DEmiRNAs between solitary and group-living silkworms, Figure S10: A to C are the GO enrichment results of DEGs in brain, midgut and cuticle, respectively, Figure S11: Statistics on microRNA sequencing data, Figure S12: Statistics of the count of reads of microRNA sequencing of the brain, Figure S13: Statistics of the count of reads of microRNA sequencing of the silkworm body with brain excised, Table S1: The ratios of molted silkworms at a time point at the molting stage after the 4th instar and before the 5th instar, Table S2: A test on the selection bias of silkworms to odors, Table S3: The comparison of the counts of behaviors between solitary and group-living silkworms in the experiment to test memory strength, Table S4: The analysis results based on the data in Table S3, Table S5: DEGs between solitary and group-living silkworms in the brain, Table S6: DEGs between solitary and group-living silkworms in the midgut, Table S7: DEGs between solitary and group-living silkworms in the cuticle, Table S8: DEmiRNAs between solitary and group-living silkworms in the brain, Table S9: DEmiRNAs between solitary and group-living silkworms in the body with brain excised.
