# Supplementary material for: Solitary Living Brings a Decreased Weight and an Increased Agility to the Domestic Silkworm, Bombyx mori
Source: Insects. 2021 Sep 9;12(9):809. doi: 10.3390/insects12090809 (PMC8470633; doi:10.3390/insects12090809)
Supplement: Supplementary file 1 [file insects-12-00809-s001.zip › Supplementary Files/Supplementary figures.pdf]

Solitary Living Brings a Decreased Weight and an Increased Agility to the Domestic Silkworm, *Bombyx mori*

Zhenglin Zhu[1*#], Yuting Tan[1#], Siyu Xiao[1#], Zhufen Guan[1#], Wen Zhao[1], Zhijun Dai[1], Gexin Liu[1], Ze Zhang[1*]

#These authors contribute equally

1. Laboratory of Evolutionary and Functional Genomics, School of Life Sciences, Chongqing University, Chongqing 401331, China

Zhenglin Zhu, Laboratory of Evolutionary and Functional Genomics, School of Life Sciences, Chongqing University, Chongqing 401331, China

TEL: (86)23-6512-2686, FAX: (86)23-6512-2689, zhuzl@cqu.edu.cn

Ze Zhang, Laboratory of Evolutionary and Functional Genomics, School of Life Sciences, Chongqing University, Chongqing 401331, China

TEL: (86)23-6512-2686, FAX: (86)23-6512-2689, zezhang@cqu.edu.cn

Keywords: solitary; loneliness; group-living; weight; agility

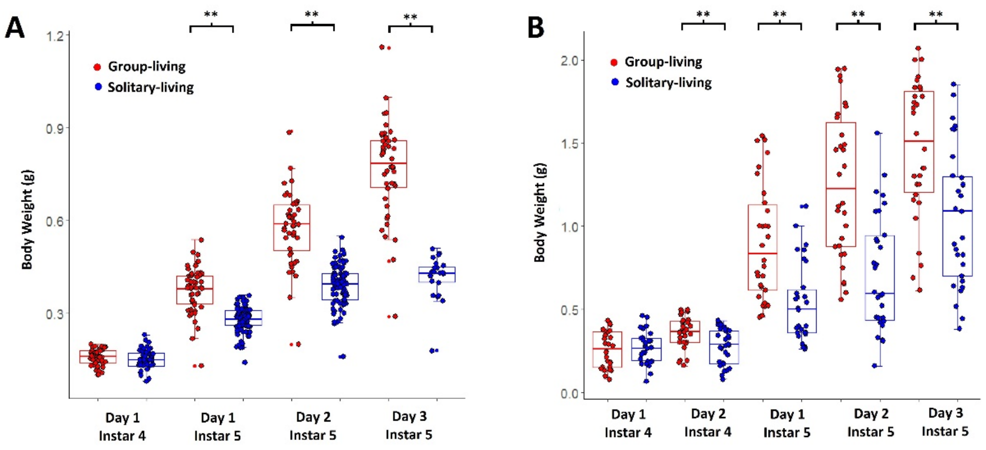

Figure S1. Repeated experiments to compare the body weight between solitary and group-living silkworms from the first day to the 3rd day of the 5th instar. In the first day of the 4th instar, we began to breed solitary silkworms. A and B are two independent results. In B, the data at the 2nd day of the 4th instar 4 is added. '*' denotes P-value < 0.05 and '**' denotes P-value < 0.01. Statistics are performed by Wilcox test.

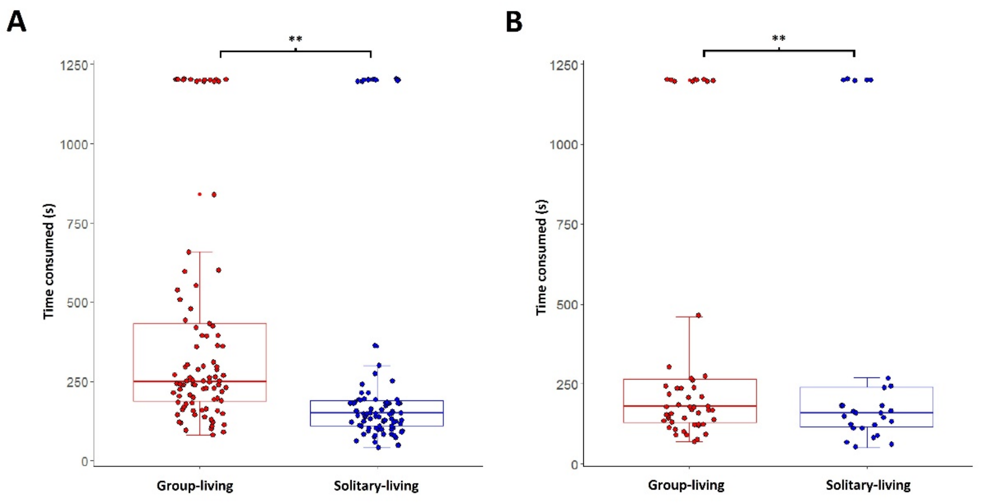

Figure S2. Repeated experiments to compare the time consumed to move under food luring between solitary and group-living silkworms. A and B are two independent results. '*' denotes P-value < 0.05 and '**' denotes P-value < 0.01. Statistics are performed by Wilcox test.

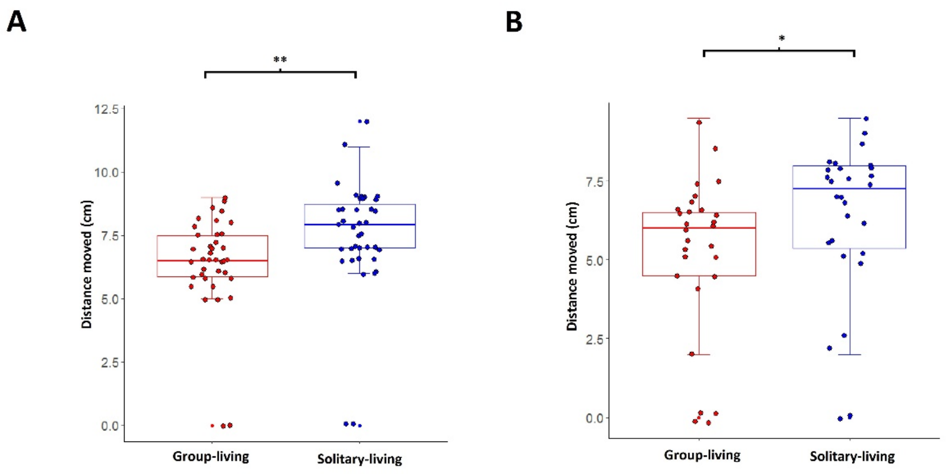

Figure S3. Repeated experiments to compare the distance moved under heat stress between solitary and group-living silkworms. A and B are two independent results. '*' denotes P-value < 0.05 and '**' denotes P-value < 0.01. Statistics are performed by Wilcox test.

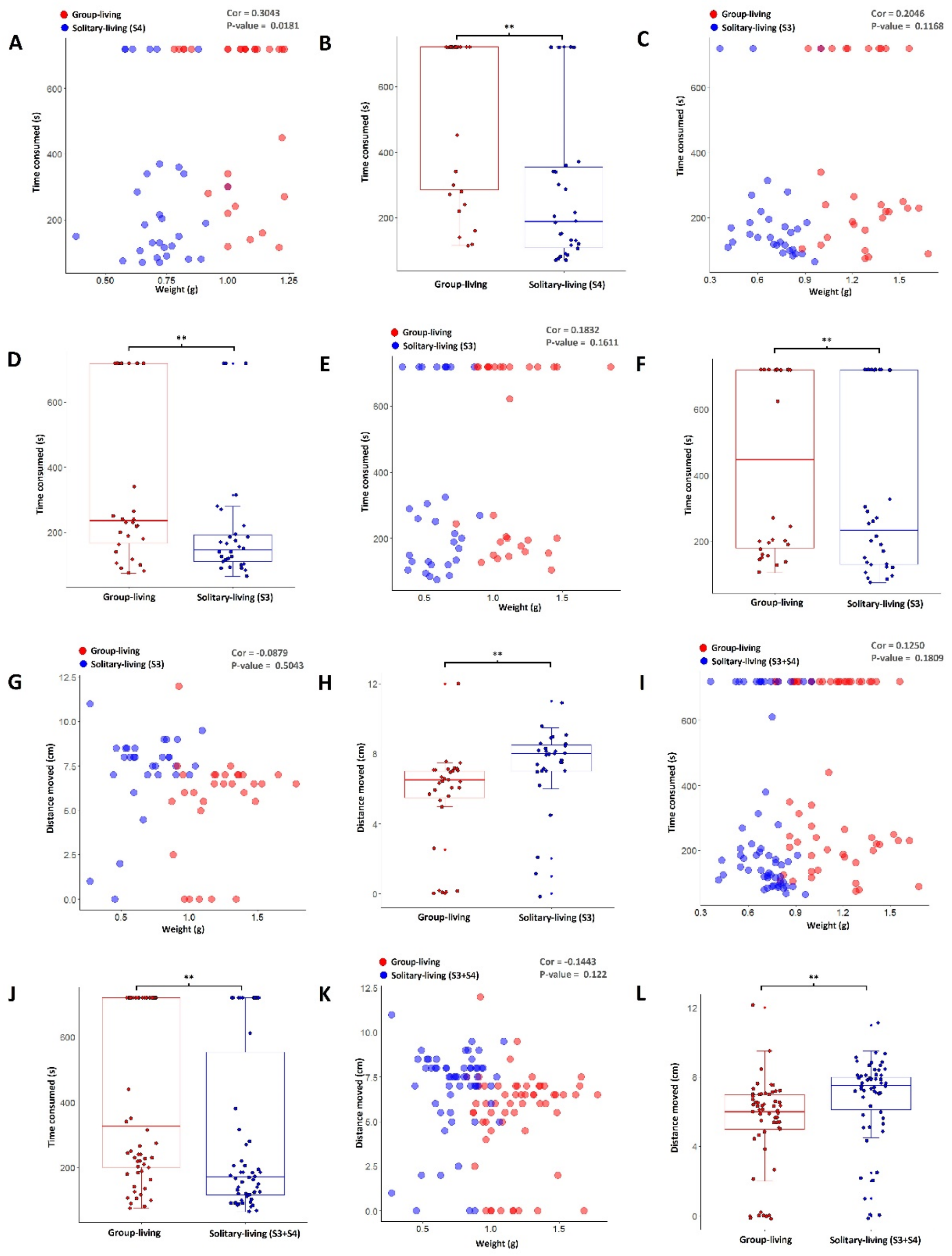

Figure S4. Repeated experiments of the correlation analysis between the weight of solitary/group-living silkworms and the performance under food luring/heat stress. A, C, E and I (mutually independent) are scatterplots showing the correlations of the time consumed to move under food and the body weight, for solitary (blue) and group-living silkworms (red). G and K are scatterplots showing the correlations of the distance moved to evade heat stress and the body weight, for

solitary (blue) and group-living silkworms (red). B, D, F and J are the time consumed to moving under food luring (corresponding to A, C, E and I, respectively) between solitary- and group-living silkworms. H and L are the distances moved (corresponding to G and K, respectively) to evade heat stress between solitary- and group-living silkworms. A and B are comparisons between group- and S4 solitary-living silkworms. C to H are comparisons between group- and S3 solitary-living silkworms. I to L are comparisons between group-living and both types of solitary silkworms, including S3 and S4. I and J are comparisons for the sum of the data in Figure 4A and the data in A to F of this figure. K and L are comparisons for the sum of the data in Figure 4B and the data in G and H of this figure. Other legends follow Figure 4. In B, D, F, H, J and L, '*' denotes P-value < 0.05 and '**' denotes P-value < 0.01. Statistics are performed by Wilcox test.

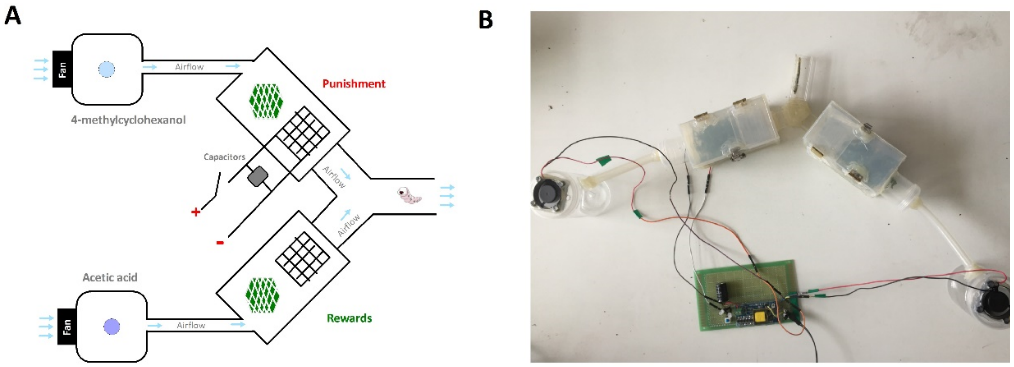

Figure S5. The apparatus used to test the memory strengths of silkworms. A is a sketch showing the details of the apparatus. B is a picture of a real product of A.

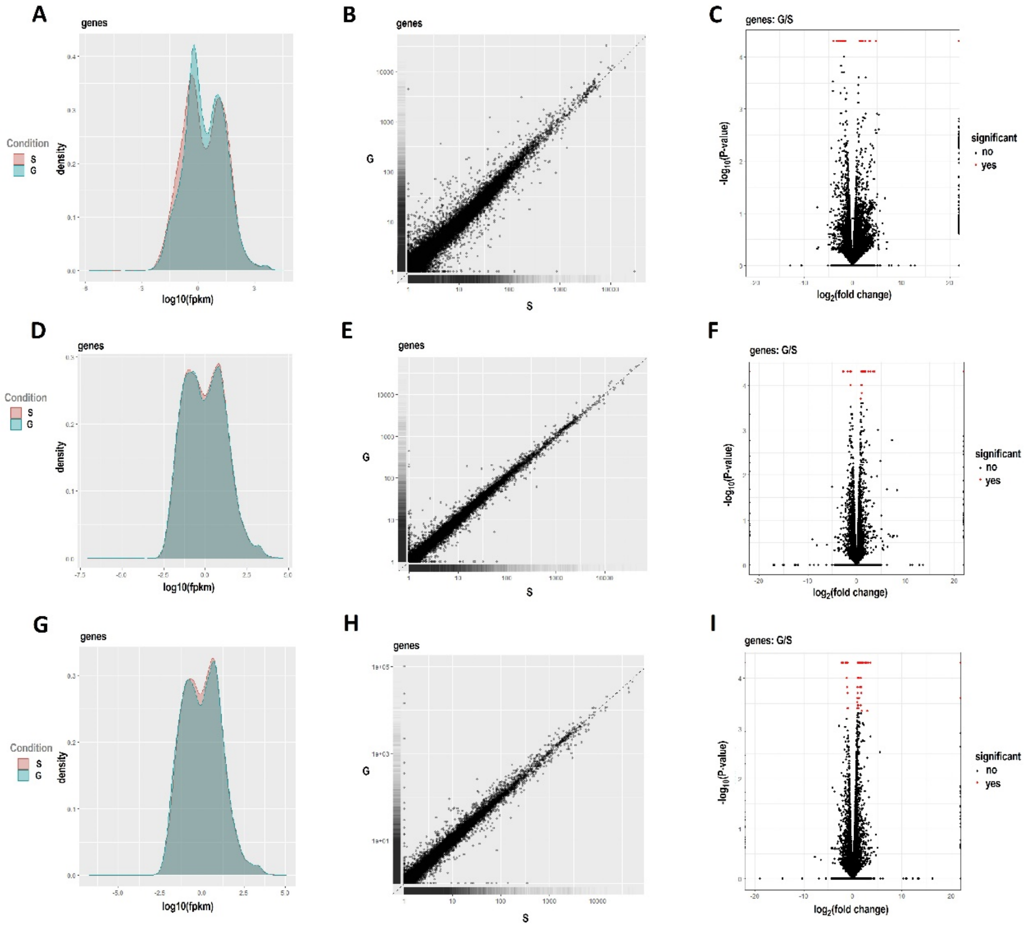

Figure S6. Statistics of RNA-seq data. A, D and G are the density plots for log10-transformed FPKM values between solitary (S) and group-living silkworms (G). B, E and H are scatter plots showing the comparison of the FPKM values between solitary (S) and group-living silkworms (G). C, F and I are volcano plots of log fold change in expression vs –log (pval) for a pair of samples (x,y), solitary (S) and group-living (G) silkworms. A, B and C are the analyses in the brain. D, E and F are the analyses in the midgut. G, H and I are the analyses in the cuticle.

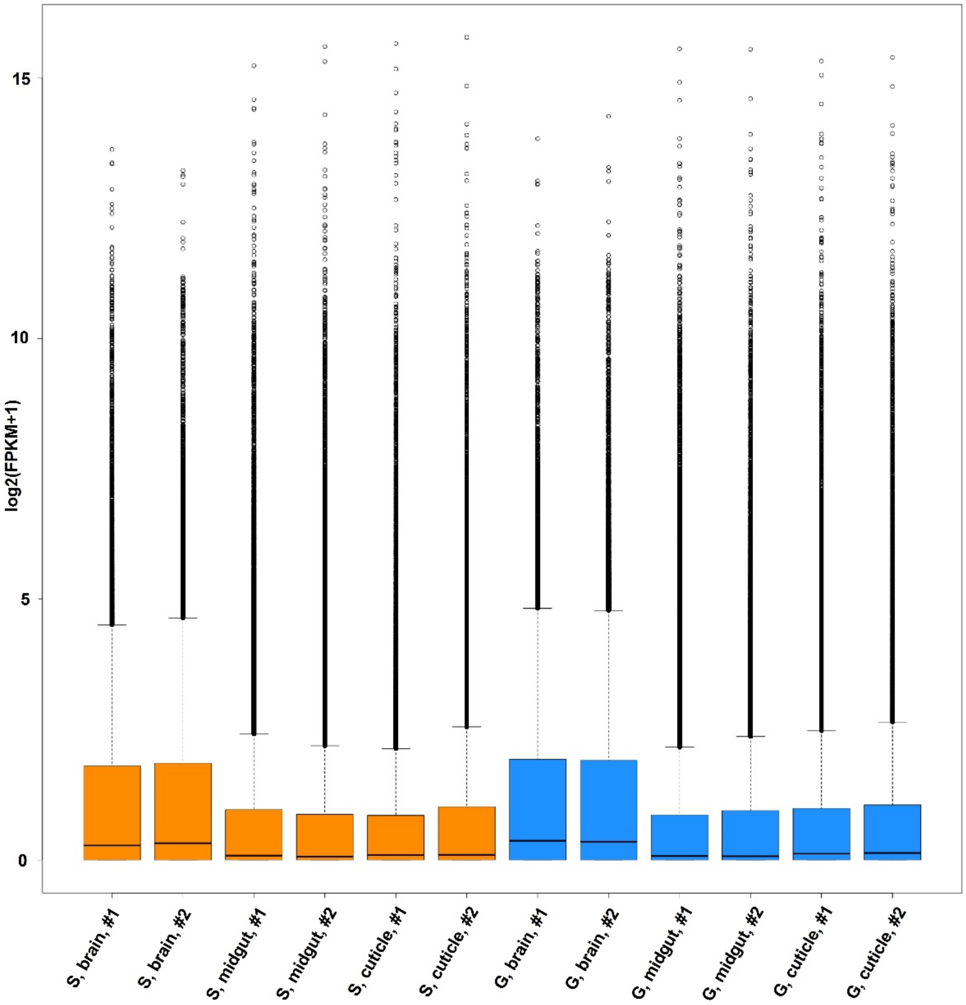

Figure S7. The distribution of genes' express log2 (FKPM+1) in tissues, including brain, midgut and brain. S and G denote solitary and group-living silkworms, respectively. '#1' and '#2' denotes the serial of two repeated sequencing data, respectively.

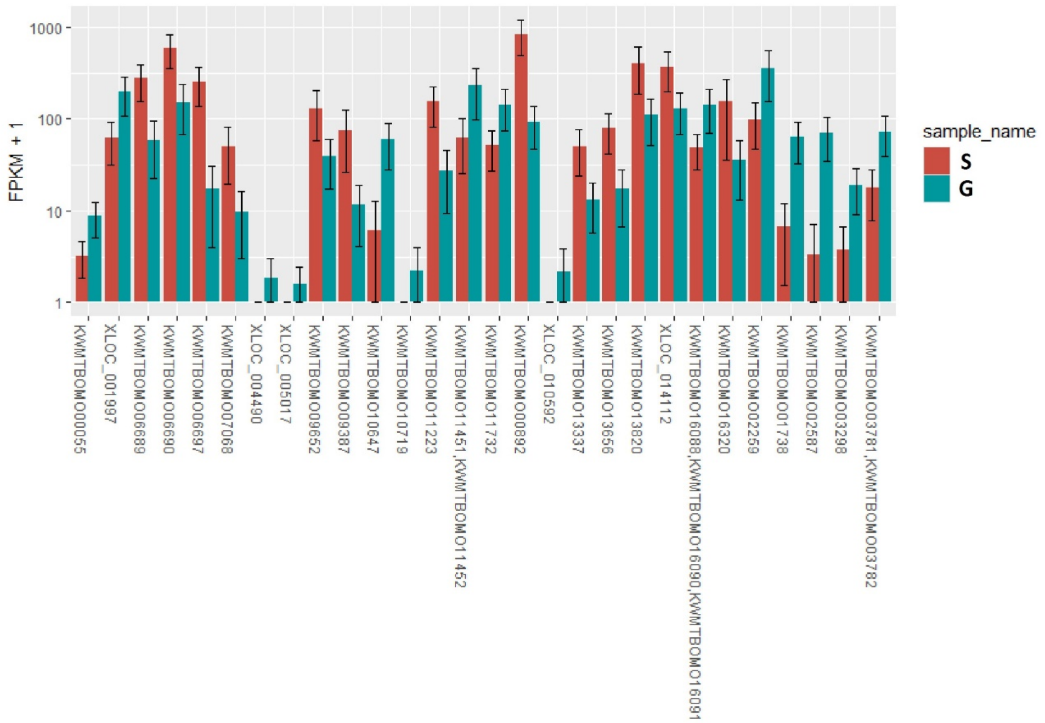

Figure S8. The expression (FKPM) of DEGs in the brain between solitary (S) and group-living (G) silkworms.

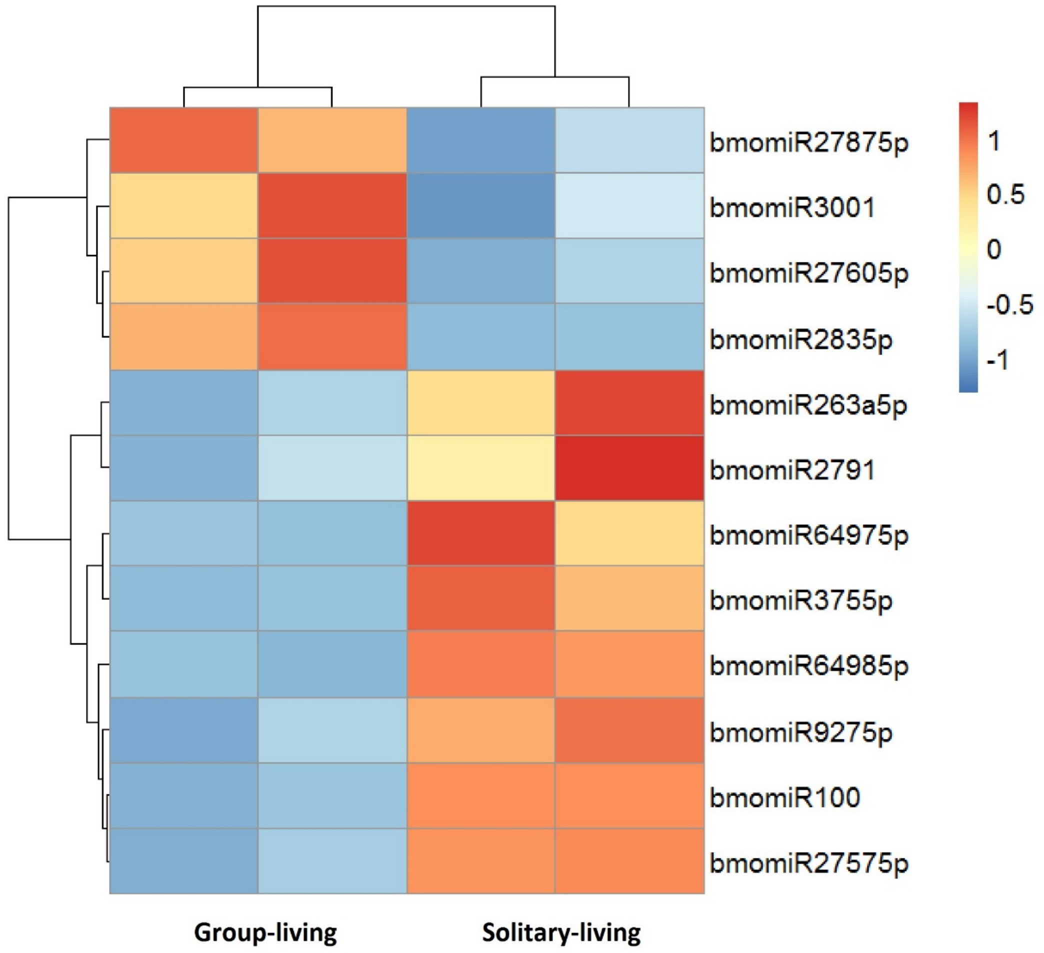

Figure S9. The heatmap of the expression (normalized) of DEmiRNAs (adjusted P-value < 0.05) between solitary and group-living silkworms.

A

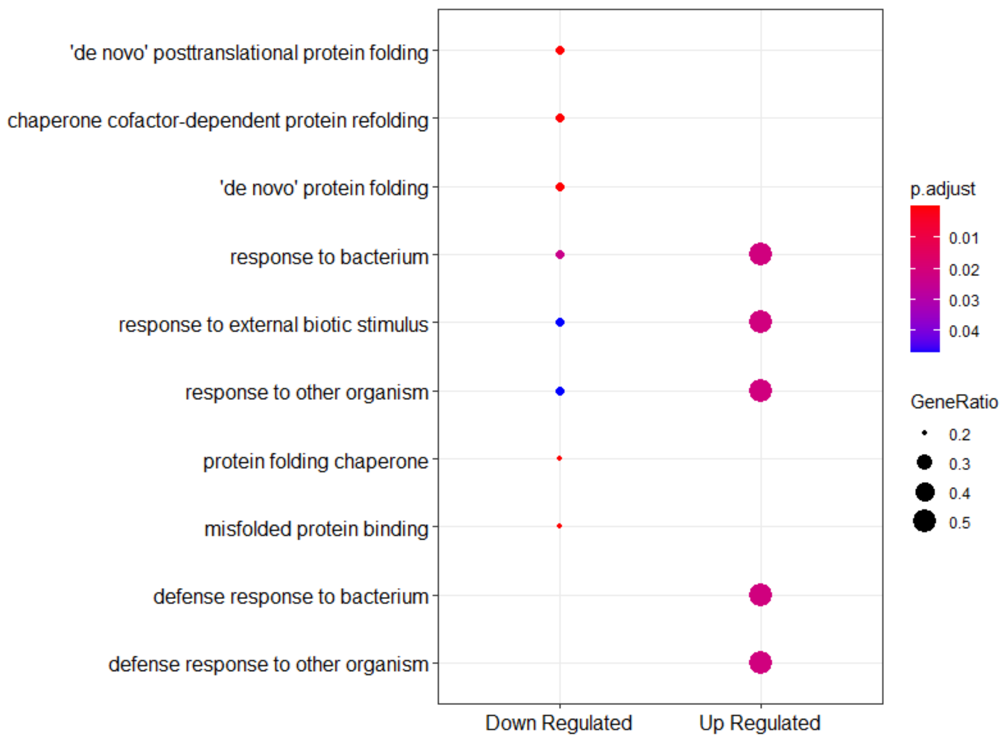
'de novo' posttranslational protein folding
chaperone cofactor-dependent protein refolding
'de novo' protein folding
response to bacterium
response to external biotic stimulus
response to other organism
protein folding chaperone
misfolded protein binding
defense response to bacterium
defense response to other organism
Down Regulated
Up Regulated
p.adjust
0.01
0.02
0.03
0.04
GeneRatio
0.2
0.3
0.4
0.5

B

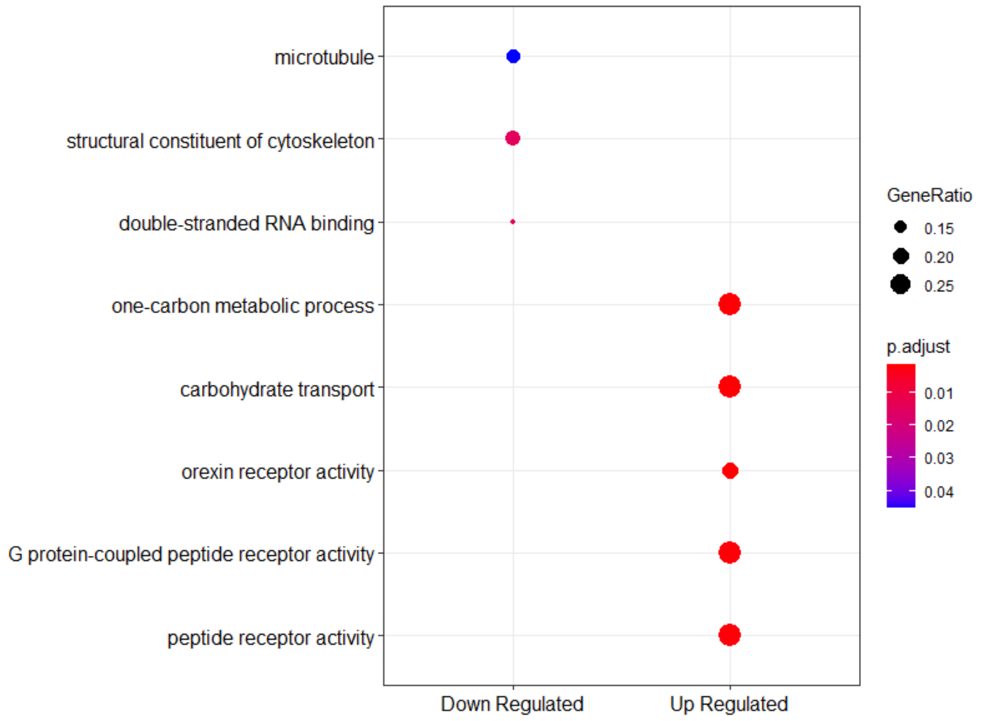
microtubule
structural constituent of cytoskeleton
double-stranded RNA binding
one-carbon metabolic process
carbohydrate transport
orexin receptor activity
G protein-coupled peptide receptor activity
peptide receptor activity
Down Regulated
Up Regulated
GeneRatio
0.15
0.20
0.25
p.adjust
0.01
0.02
0.03
0.04

C

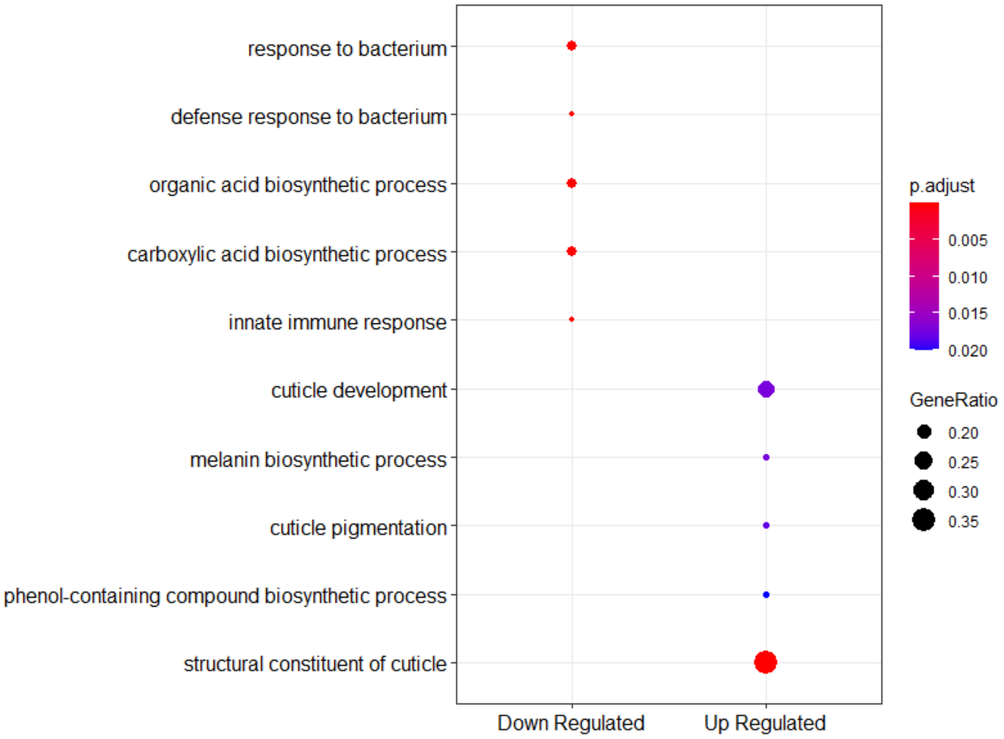

Figure S10. A to C are the GO enrichment results of DEGs in brain, midgut and cuticle, respectively. 'p.adjust' denotes the adjusted P-value of enriched pathways.

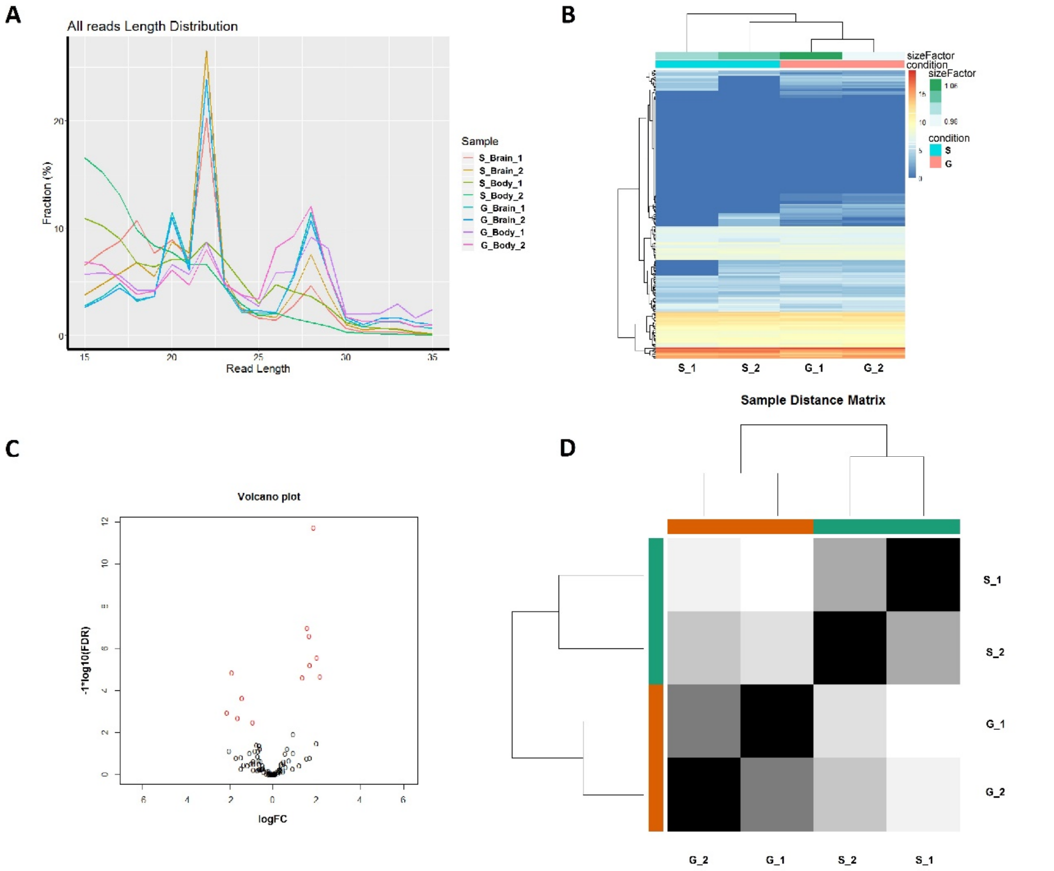

Figure S11. Statistics on microRNA sequencing data. A is all reads length distribution in the brain and the body (with brain excised) of solitary (S) and group-living (G) silkworms. B is the heatmap of all miRNAs with expression signals in the brain of solitary (S) and group-living (G) silkworms. C is a volcano plot of log fold change in expression vs –log (pval) for a pair of samples (x,y), the brains of group and solitary-living silkworms. miRNA with -1*log10(FDR)<0.01 are in red. D is the sample distance matrix of the experiments of the brain of solitary (S) and group-living (G) silkworms. In A, B and D, 1 and 2 denote the serials of two repeated experiments.

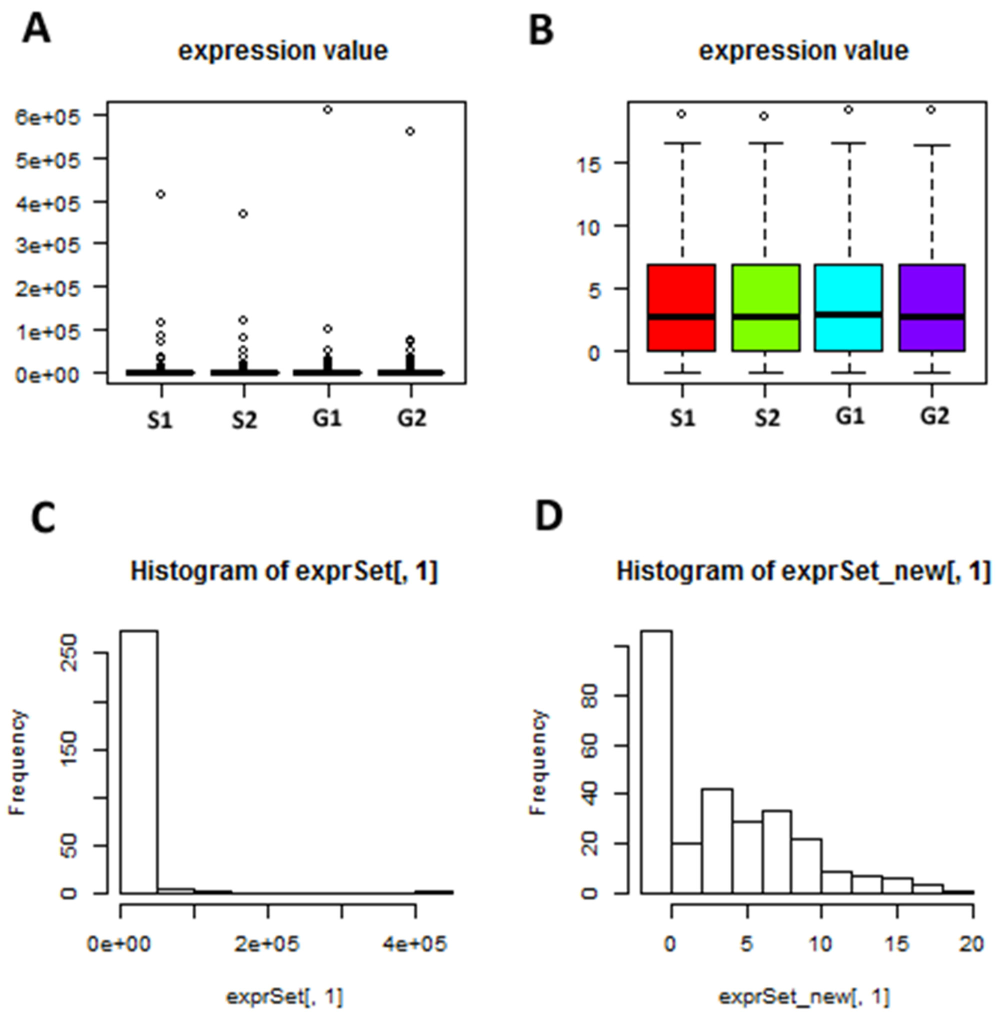

Figure S12. Statistics of the count of reads of microRNA sequencing of the brain. A and B correspond to the distribution of the original (A) and normalized expression (B) values in solitary (S) and group-living (G) silkworms. C is the histogram of the expression of all reads. D is the histogram of the expression normalized of all reads. In A and B, 1 and 2 denote the serials of two repeated experiments.

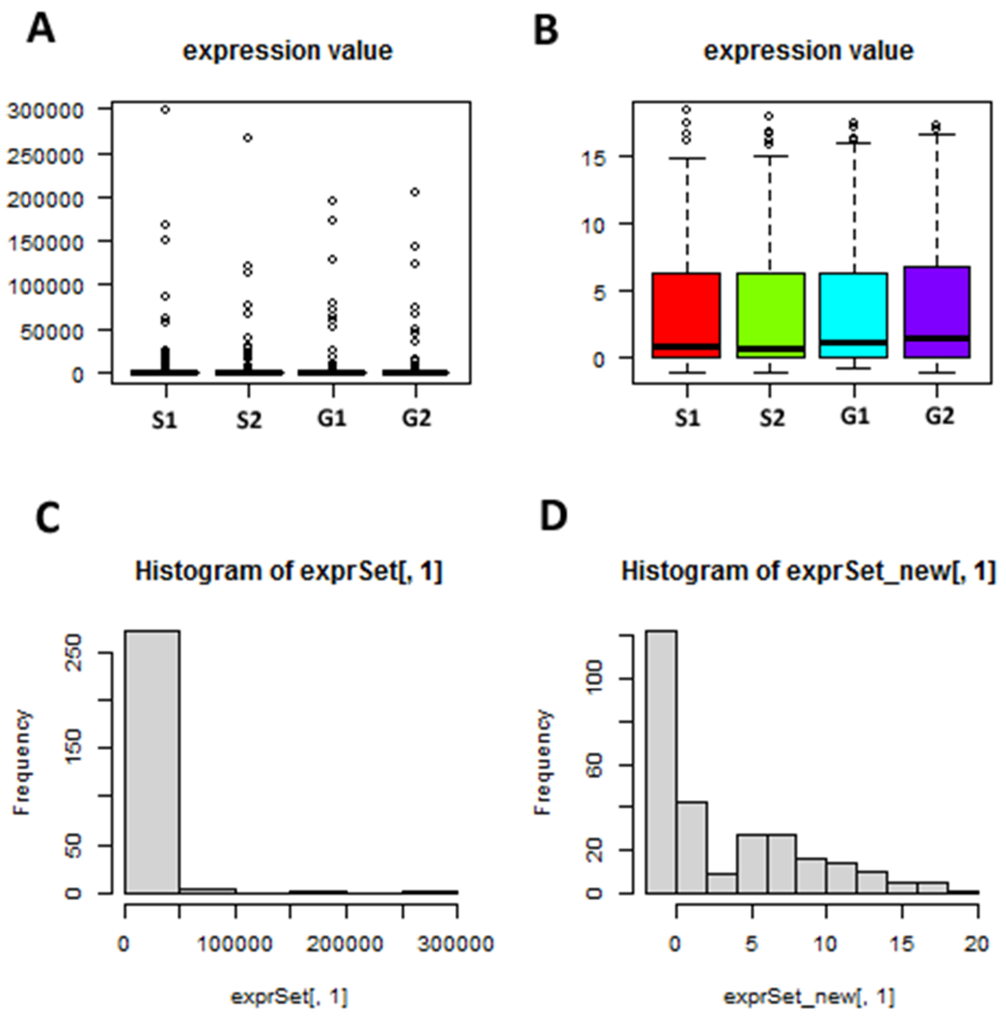

Figure S13. Statistics of the count of reads of microRNA sequencing of the silkworm body with brain excised. The figure convention in A to D follows Figure S9.
